# Inflammatory cytokines and immune signalling in heart failure: mechanisms, models, and emerging therapies

**DOI:** 10.1093/cvr/cvag172

**Published:** 2026-08-18

**Authors:** Guiomar Mendieta, Gianluigi Pironti, Jasper Tromp, Lars H Lund

**Affiliations:** Department of Cardiology, Hospital de la Santa Creu i Sant Pau, Institut de Recerca Sant Pau (IR SANT PAU), CIBERCV, Universitat Autònoma de Barcelona, Carrer de Sant Quintí, 89, Barcelona 08025, Spain; Centro Nacional de Investigaciones Cardiovasculares Carlos III (CNIC), Madrid, Spain; Department of Physiology and Pharmacology, Karolinska Institutet, Stockholm, Sweden; Department of Medicine, Cardiology Unit, Karolinska Institutet, Stockholm, Sweden; Department of Cardiology, Karolinska University Hospital, Stockholm, Sweden; Yong Loo Lin School of Medicine, National University of Singapore, Singapore; Saw Swee Hock School of Public Health & the National University Health System, National University of Singapore, Singapore; Department of Medicine, Cardiology Unit, Karolinska Institutet, Stockholm, Sweden; Department of Cardiology, Karolinska University Hospital, Stockholm, Sweden

**Keywords:** Animal models, Comorbidities, Heart failure, High-sensitivity C-reactive protein, IL-6

## Abstract

Inflammation plays a central role in the pathophysiology of heart failure (HF), and contributes to disease progression, morbidity, and mortality. However, the relationships between inflammatory cytokines and different HF subtypes are complex, heterogeneous, and bidirectional. In this review, we examine inflammatory pathways, biomarker profiles, and comorbidities in HF with reduced, mildly reduced, and preserved ejection fraction and summarize evidence on the use of high-sensitivity C-reactive protein as a biomarker for systemic inflammation in HF. We provide insights into emerging methodologies for biomarker discovery in HF and discuss the use of animal models to elucidate underlying mechanisms and test potential treatment strategies. Finally, we provide an overview of the therapeutic landscape, touching on past challenges and future opportunities for the development of targeted anti-inflammatory therapies in HF.

**Time of primary review: 22 days**

## 1. Introduction

Heart failure (HF) affects 1–3% of adults in the USA and Europe, and mortality is 50–75% over 5 years.^1^ HF has varying causes, pathophysiology, and clinical manifestations across different phenotypes. Coronary artery disease and hypertension are the most common causes of HF globally, although the aetiology of HF varies geographically.^2,3^ Subtypes of HF are defined by left ventricular ejection fraction (LVEF) as follows:^4^

- HF with reduced ejection fraction (HFrEF)—HF with LVEF ≤ 40%
- HF with mildly reduced ejection fraction (HFmrEF)—HF with LVEF 41–49%
- HF with preserved ejection fraction (HFpEF)—HF with LVEF ≥ 50%.

The prevalence of each HF subtype varies across populations and settings. In referral populations, HFrEF predominates, whereas in population-based cohorts, HFpEF is more prevalent.^1,5–8^ The prevalence of HFpEF compared with HFrEF is increasing, driven by an ageing population and the increasing prevalence of comorbidities associated with HFpEF.^9^ Typically, patients with HFrEF have a higher risk of cardiovascular (CV) death and HF hospitalization (HHF) and worse quality of life than those with HFpEF.^1,10–13^

There are differences in pathophysiology between HF subtypes. HFrEF and HFmrEF are characterized by loss of cardiomyocyte contractility, resulting in systolic dysfunction.^14–16^ Diastolic dysfunction is also common in patients with HFrEF,^17^ with more severe dysfunction associated with increased risk of death.^18^ HFpEF is likely driven by inflammation, fibrosis, and post-translational modifications of cardiomyocyte proteins,^19^ leading to diastolic dysfunction and impaired ventricular filling.^20–23^ HFpEF phenotypes can be defined according to pathological, structural, and/or haemodynamic signs, by the presence of specific comorbidities and risk factors, or by response to therapy.^20,24,25^ Whereas HFpEF and HFrEF arise from different pathophysiological mechanisms, HFmrEF has features of both but most closely resembles HFrEF.^8^ HFpEF is also preceded and characterized by more comorbidities.^14,15^ For example, chronic kidney disease (CKD), chronic pulmonary disease, and valvular heart disease are more common in HFpEF than in HFrEF. In contrast, hyperlipidaemia, peripheral artery disease, and cerebrovascular disease may be more common in HFrEF than in HFpEF.^26^ HFpEF is also strongly linked to hypertension^27^ and obesity.^28^ Most distinctly, HFrEF and HFmrEF are both considerably more associated with ischaemic heart disease (IHD) than HFpEF.^8,29,30^

Inflammation is thought to play a central role in the pathophysiology of all subtypes of HF,^31,32^ and elevated circulating inflammatory markers are associated with both the occurrence of incident HF and worse outcomes in prevalent HF.^33,34^ However, the role of inflammation in HF varies across the spectrum of LVEF. Unlike in HFrEF, in which inflammation results from myocardial injury and remodelling, in HFpEF, the chronic pro-inflammatory state driven by multiple comorbidities causes endothelial dysfunction, fibrosis, and vascular stiffening, which impair diastolic function and result in HF symptoms.^35^ Certain inflammatory conditions, such as rheumatoid arthritis (RA), are also independently associated with HF, and more commonly with HFpEF than HFrEF.^36,37^ The relationship between HF and inflammation is bidirectional. Although chronic HF is associated with circulating inflammatory cytokines that can predict clinical outcomes, inflammation may also be a driving factor in the aggravation and progression of HF.^38^  *Figure 1* shows a simplified overview of the pathways and processes implicated in the different HF subtypes.

**Figure 1:**
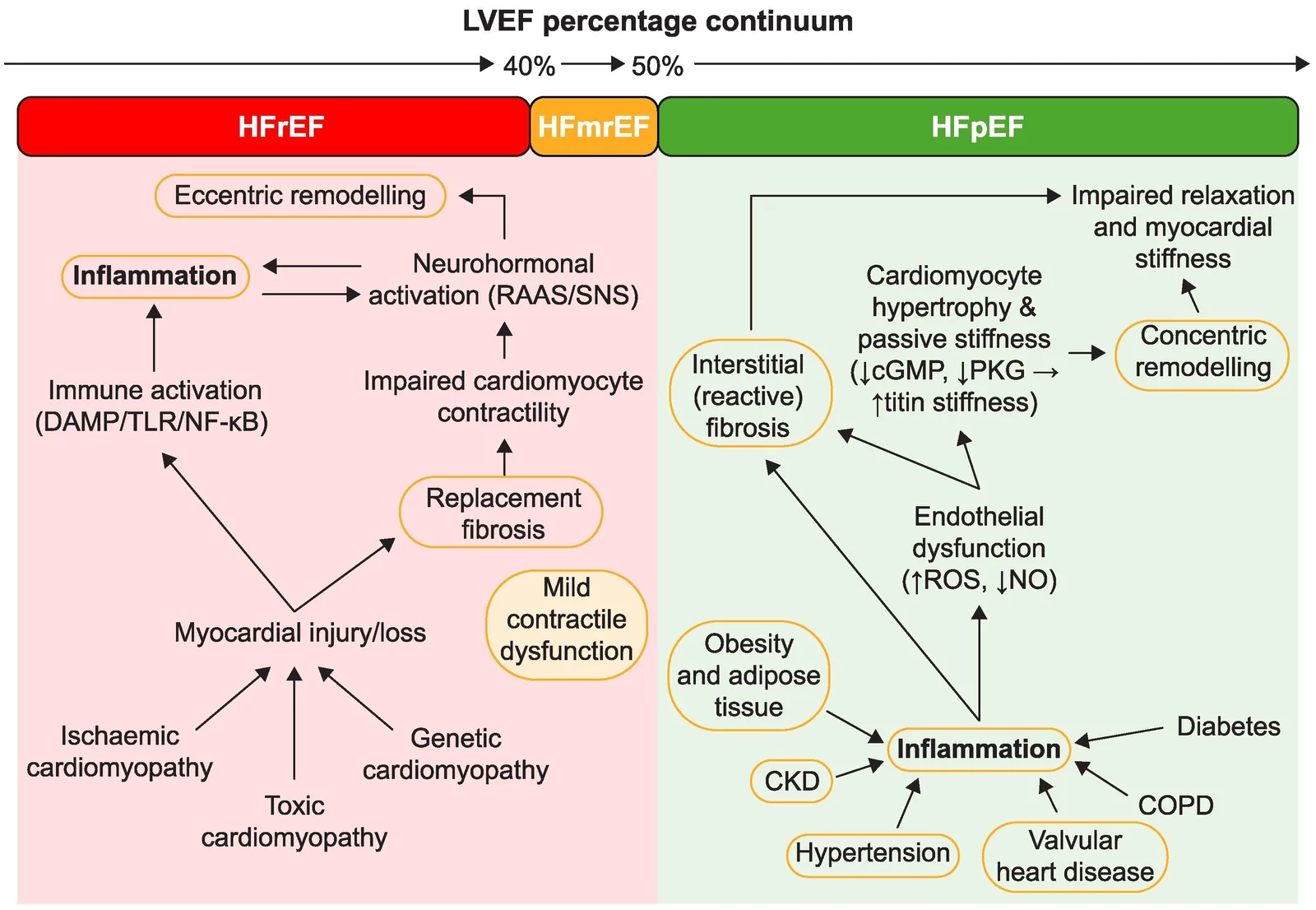
Simplified overview of pathophysiological processes in HF subtypes and aetiologies. Adapted in part from Paulus *et al.* A novel paradigm for heart failure with preserved ejection fraction: comorbidities drive myocardial dysfunction and remodelling through coronary microvascular endothelial inflammation. *J Am Coll Cardiol* 2013; 62:263–71.^39^ CKD, chronic kidney disease; cGMP, cyclic guanosine monophosphate; COPD, chronic obstructive pulmonary disease; DAMP, damage-associated molecular pattern; HFmrEF, heart failure with mildly reduced ejection fraction; HFpEF, heart failure with preserved ejection fraction; HFrEF, heart failure with reduced ejection fraction; LVEF, left ventricular ejection fraction; NF-κB, nuclear factor kappa B; NO, nitric oxide; PKG, protein kinase G; RAAS, renin–angiotensin–aldosterone system; ROS, reactive oxygen species; SNS, sympathetic nervous system; TLR, Toll-like receptor.

The pathophysiological distinction between HFpEF, HFmrEF, and HFrEF is reflected in differences in guideline-directed medical therapy (*Table 1*). In both European^3^ and US^40^ treatment guidelines, the cornerstone therapy to improve survival and reduce HHF for patients with HFrEF consists of angiotensin-converting enzyme inhibitors (ACEIs) or angiotensin receptor neprilysin inhibitors (ARNIs), beta-blockers, and mineralocorticoid receptor antagonists (MRAs). A sodium-glucose cotransporter-2 inhibitor (SGLT2i; dapagliflozin or empagliflozin) is recommended as an add-on to reduce the risk of CV death and worsening HF.^3,40^ In contrast, the guideline-directed medical therapy for HFpEF and HFmrEF is less well established. In the 2023 focused update of the European Society of Cardiology guidelines, SGLT2is were recommended to reduce the risk of CV death and HHF (class 1 recommendation)^41^; similarly, in 2022, the American Heart Association, American College of Cardiology, and Heart Failure Society of America (AHA/ACC/HFSA) suggested that SGLT2is can be beneficial for patients with HFpEF or HFmrEF (class 2a).^40^ ACEIs/ARNIs, beta-blockers, and MRAs may be used, but the supporting evidence is less robust (class 2b).^3,40,41^

**Table 1:** Guideline-directed medical therapy according to HF subtype

	HFpEF	HFmrEF	HFrEF
**ACEI**	Sometimes (class 2b)^40^	Sometimes (class 2b)^3,40,41^	Yes (class 1)^3,40,41^
**ARNI**	Sometimes (class 2b)^40^	Sometimes (class 2b)^3,40,41^	Yes (class 1)^3,40,41^
**Beta-blockers**	Sometimes (class 2b)^40^	Sometimes (class 2b)^3,40,41^	Yes (class 1)^3,40,41^
**MRA**	Sometimes (class 2b)^40^	Sometimes (class 2b)^3,40,41^	Yes (class 1)^3,40,41^
**SGLT2i**	Yes (class 1/class 2a)^3,40,41^	Yes (class 1/class 2a)^3,40,41^	Yes (class 1)^3,40,41^

ACEI, angiotensin-converting enzyme inhibitor; ARNI, angiotensin receptor neprilysin inhibitor; HF, heart failure; HFmrEF, heart failure with mildly reduced ejection fraction; HFpEF, heart failure with preserved ejection fraction; HFrEF, heart failure with reduced ejection fraction; MRA, mineralocorticoid receptor antagonist; SGLT2i, sodium-glucose cotransporter-2 inhibitor.

As described, from a pathophysiological perspective, HFmrEF appears to occupy an intermediate position between HFpEF and HFrEF, with overlapping mechanisms of myocardial injury, remodelling, and neurohormonal activation. However, in contrast to HFpEF and HFrEF, the specific role of inflammation in HFmrEF remains incompletely characterized. To date, most available data derive from post hoc analyses or extrapolation from adjacent ejection fraction (EF) categories, and dedicated studies exploring inflammatory biomarkers, immune cell profiles, or inflammatory signalling pathways in HFmrEF are scarce. As such, while HFmrEF is increasingly recognized as a clinically relevant phenotype, further targeted research is required to define its inflammatory signature and the potential implications for risk stratification and therapeutic targeting. Accordingly, in the present review, we focus primarily on HFpEF and HFrEF, for which the evidence base regarding inflammation is more robust, while acknowledging HFmrEF as an important area for future investigation.

In this review, we use ‘systemic inflammation’ primarily to refer to circulating inflammatory cytokines and other biomarkers, such as C-reactive protein (CRP) and interleukin (IL)-6, rather than inflammatory signalling intrinsic to cardiac cells. We describe the heterogeneity of inflammatory pathways, comorbidities, and manifestations in HF subtypes. Further, we highlight novel approaches for biomarker discovery, and discuss inflammatory cytokines in preclinical animal models of HF, treatments targeting inflammatory pathways, and the outlook for new emerging treatments.

## 2. Inflammatory pathways and cytokines in HF

### 2.1 Key inflammatory pathways

Multiple inflammatory pathways contribute to HF pathophysiology by coordinating the activation of both innate and adaptive immunity systems. The former offers a broad, non-specific response to pathogens and/or tissue damage, while the latter mounts targeted, antigen-specific responses through B- and T-cell activation.

One of these pathways is the nucleotide-binding oligomerization domain-like receptor family pyrin domain-containing 3 (NLRP3) inflammasome (*Figure 2*). Priming and activation of the NLRP3 inflammasome can be triggered by various pathological conditions arising from cardiovascular disease (CVD) or comorbidities.^42,43^ The main factors implicated in initiation of the process relate to haemodynamic stress (mechanical stress or ischaemia), metabolic stress or oxidative or inflammatory stress (the presence of ROS or pathogens).^43^ Such triggers affect a broad range of different cell types. In brief, cytokines, such as tumour necrosis factor (TNF)-α and IL-1β, or toll-like receptor (TLR) signalling lead to transcription of NLRP3 inflammasome components and release of pro-IL-1β.^43^ Assembly and activation of the NLRP3 inflammasome may be triggered by lysosomal damage or the presence of particulate matter, such as uric acid or cholesterol crystals, mitochondrial dysfunction, or potassium ion flux owing to the action of adenosine triphosphate.^43–45^ Upon activation, the proteinase caspase-1 contained in the NLRP3 inflammasome cleaves pro-IL-18 and pro-IL-1β into their active forms, IL-18 and IL-1β, via pyroptosis, which triggers pro-inflammatory signalling via IL-6.^43,45,46^ Among other downstream effects, IL-6 activation triggers the hepatic production of CRP.^46^ IL-6 levels are a predictor of incident HFpEF,^47,48^ as well as all-cause mortality, CV death, HHF, and worsening disease severity in patients with HF.^49–51^ This relationship between IL-6 levels and poor outcomes has been observed separately in HFpEF^51^ and HFrEF.^49^

**Figure 2:**
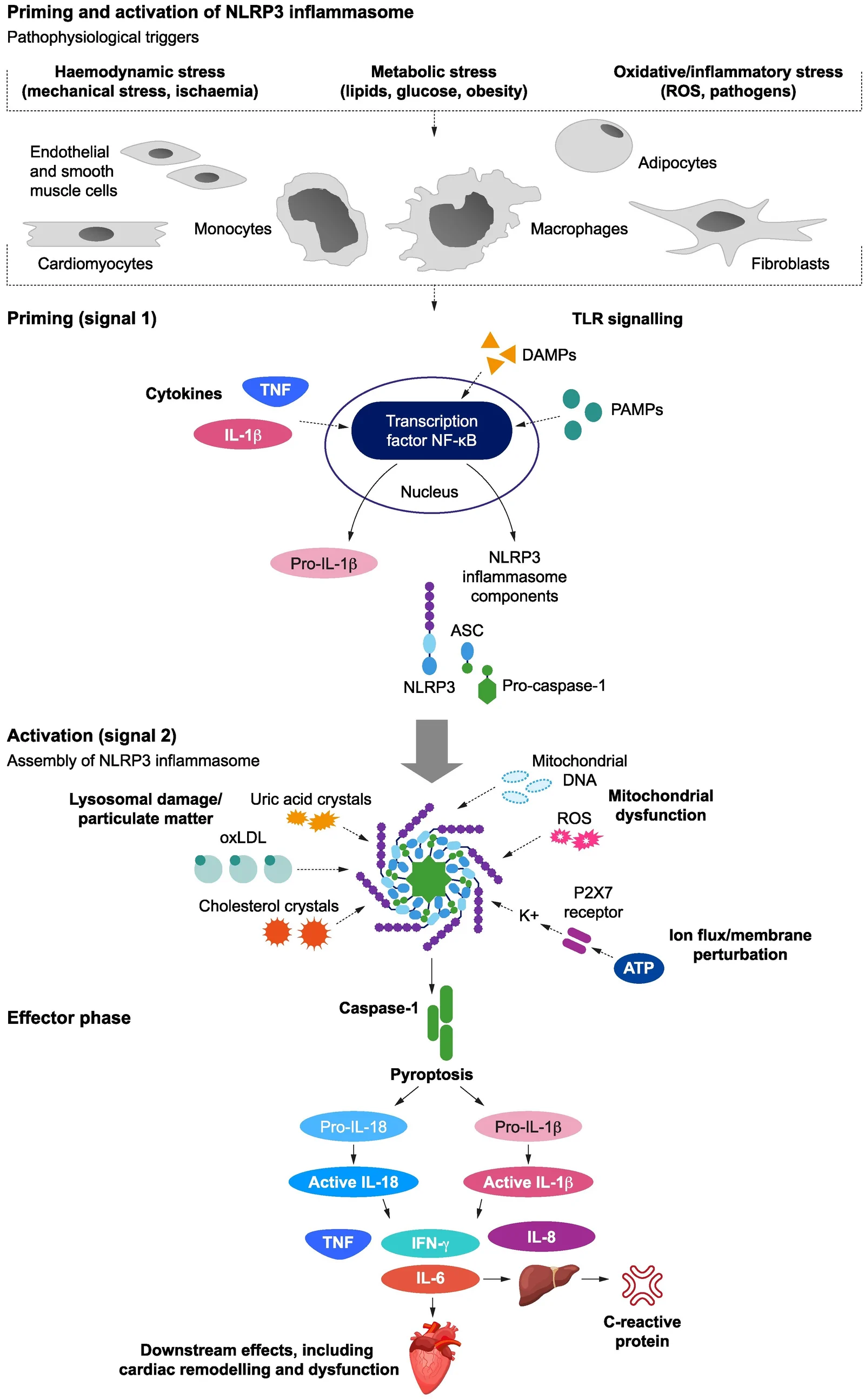
Priming and activation of the NLRP3 inflammasome: pathophysiological triggers, cell types implicated, and factors that lead to priming and release of inflammatory cytokines. ASC, apoptosis-associated speck-like protein containing a caspase recruitment domain; ATP, adenosine triphosphate; DAMP, damage-associated molecular pattern; IFN-γ, interferon-gamma; IL, interleukin; NF-κB, nuclear factor kappa-light-chain-enhancer of activated B cells; NLRP3, nucleotide-binding domain, leucine-rich containing family pyrin domain containing 3; oxLDL, oxidized low-density lipoprotein; PAMP, pathogen-associated molecular pattern; P2X7, purinergic receptor P2X7; ROS, reactive oxygen species; TLR, Toll-like receptor; TNF, tumour necrosis factor.

Several other key inflammatory pathways contribute significantly to HF pathophysiology and provide distinct therapeutic targets. TLR4 serves as the primary sensor for damage-associated molecular patterns, driving sustained inflammatory activation through nuclear factor-κB (NF-κB) signalling, which promotes cardiomyocyte apoptosis and adverse remodelling.^52,53^ The NF-κB pathway acts as the central transcriptional hub orchestrating inflammatory gene expression, with dual cardioprotective and pathological functions depending on activation timing.^54^ Janus kinase/signal transducer and activator of transcription (JAK-STAT) signalling mediates cytokine responses with opposing roles: STAT3 provides cardioprotection, whereas STAT5 promotes pathological remodelling.^55,56^ The complement system activates through classical, lectin, and alternative pathways, generating C5a anaphylatoxin and membrane attack complexes that drive acute cardiac injury and chronic remodelling.^57,58^ Novel biomarkers, including galectin-3 (reflecting cardiac fibrosis), soluble ST2 (IL-33/ST2L pathway disruption), pentraxin-3 (early inflammatory activation), and myeloperoxidase (neutrophil activation), provide pathway-specific insights for targeted therapeutic decisions.^59–62^

Recent single-cell and spatial transcriptomic studies establish HF as a multicellular immune-driven disease in which distinct immune cell subsets orchestrate remodelling through tightly coupled interactions with stromal cells,^63,64^ across all HF aetiologies and subtypes.^65^ Mouse models of HF indicate that immune activation occurs across the whole spectrum of immune cell types.^63^ Metabolic reprogramming of cardiomyocytes and immune cells in HF results in metabolic crosstalk between these cell types, which can result in either maladaptive remodelling or an adaptive response.^66^ Furthermore, immune cell infiltration drives mechanical and electrical remodelling through specific fibroblast-immune cell interactions.^63,64^ Infiltrating CCR2^+^ monocyte-derived macrophages replace resident cardiac macrophages and adopt pro-inflammatory phenotypes characterized by IL-1β, TNF-α, and TGF-β secretion, which directly activate specialized fibroblast populations (e.g. POSTN^+^ and FAP^+^ fibroblasts) to promote extracellular matrix deposition and electrical remodelling.^67,68^ This process is amplified by maladaptive T cell responses, with expansion of Th1 and Th17 subsets and relative deficiency of regulatory T cells sustaining macrophage activation and fibroblast reprogramming through cytokine-mediated feedback loops.^63,65^ These immune–stromal interactions form the central axis of HF progression and differ across phenotypes, with inflammatory macrophage infiltration dominating post-infarction HFrEF, and chronic low-grade immune activation coupled to microvascular dysfunction characterizing HFpEF.^65,69^ Cross-talk between macrophages and fibroblasts via SPP1 and TNF-α has also been identified as a potential feature of HFpEF.^69^ Importantly, these pathways are tightly integrated with neurohormonal signalling. The renin–angiotensin–aldosterone system (RAAS), an upstream modulator of NLRP3 inflammasome activation, also creates extensive bidirectional crosstalk with inflammatory pathways, as angiotensin II directly activates macrophages and fibroblasts via AT1 receptor–mediated NF-κB signalling.^70^ Inflammatory cytokines further upregulate AT1 receptor expression, establishing a feed-forward loop that links RAAS activation to sustained immune-driven remodelling. In contrast, inflammatory cytokines upregulate AT1 receptors, creating self-perpetuating cycles of inflammation and cardiac damage.^70^ Cardiac inflammation also promotes remodelling via the transdifferentiation of fibroblasts to myofibroblasts; furthermore, fibroblast activation as a result of cardiac dilation in HF triggers inflammatory pathways.^71^ Interaction between fibroblasts and immune cells has been identified as a possible therapeutic target: an *in vivo* study found that inhibition of IL-1β attenuated fibroblast differentiation into disease-associated cell types, limiting myocardial fibrosis and improving cardiac function.^67,72^ Therapeutic strategies should take into account the interaction between immunological and metabolic pathways in HF,^66^ and between different cell types.^72^

### 2.2 Differing biomarker profiles across HF subtypes

Despite commonalities in biomarker elevation (e.g. natriuretic peptides) across the spectrum of HF, there are disparities in the inflammatory pathways activated across different HF subtypes. Biomarker profiles have been shown to differ between HFpEF and HFrEF,^73–75^ highlighting the involvement of differing biological processes and variations in the upregulation of common inflammatory pathways. A comparison between the HFpEF and HFrEF cohorts from the TIME-CHF trial found higher levels of soluble IL-1 receptor-like 1 (ST2), cystatin-C, and high-sensitivity C-reactive protein (hsCRP) in HFpEF relative to HFrEF, but higher levels of N-terminal pro-B-type natriuretic peptide (NT-proBNP), high-sensitivity troponin T, and haemoglobin in HFrEF.^73^ These findings were supported by a later analysis of patients from the COACH trial, in which hsCRP levels were significantly higher in HFpEF than in HFrEF, but levels of pro-atrial-type natriuretic peptide and NT-proBNP were higher in HFrEF. Linear regression and network analyses in this study indicated that the biomarker association profile of HFpEF is strongly linked to inflammation and angiogenesis, whereas biomarker interactions in HFrEF are more closely associated with cardiac stretch.^74^ Subsequently, network analyses using data from the BIOSTAT-CHF study found that integrin subunit beta-2 and catenin beta-1 were key proteins in HFpEF, whereas for HFrEF, key proteins were NT-proBNP, ST2, growth differentiation factor-15, and activating transcription factor 2. This study also identified biological pathways implicated in each HF subtype. HFpEF was linked to inflammation, cytokine response, and extracellular matrix organization. Distinct pathways related to HFrEF were DNA-binding transcription factor activity, cellular protein metabolism, and regulation of nitric oxide biosynthesis.^75^

Such analyses have also hinted at the prognostic value of specific biomarkers. In the analysis of patients from COACH, neuropilin, and osteopontin were predictive of all-cause mortality and HF-related rehospitalization in HFpEF but not HFrEF.^74^ In the Multi-Ethnic Study of Atherosclerosis, IL-6, CRP, and TNF-α levels were all associated with incident HF; however, IL-6 and CRP were associated with incident HFpEF, but not with incident HFrEF.^76^ Similarly, in the Ludwigshafen Risk and Cardiovascular Health study of patients referred for coronary angiography, IL-6 levels were predictive of CV mortality in HFpEF but not HFrEF, and the same pattern was observed for hsCRP, albeit without statistical significance.^33^ hsCRP was also among the biomarkers identified as elevated in HFpEF but not in HFrEF in a network analysis of 33 biomarkers.^74^ These observations support the comorbidity-driven systemic inflammation hypothesis as a driver specifically of HFpEF.^7,66,77–79^ However, in the Valsartan Heart Failure Trial (Val-HeFT), hsCRP levels were significantly elevated in patients with HFrEF with more advanced disease severity and congestion and provided incremental prognostic value to that provided by BNP alone.^80,81^ Elevated hsCRP concentrations were independently associated with increased mortality and morbidity, and 12-month treatment with valsartan, in patients not receiving ACEIs, was associated with a significant reduction in hsCRP levels.^80^ Although evidence regarding the prognostic utility of hsCRP has been heterogeneous, with some studies reporting no incremental value beyond established risk predictors,^82^ such discrepancies may reflect differences in patient populations and underscore the importance of clinical phenotyping. In this context, hsCRP may be particularly informative in subsets of HF characterized by a heightened systemic inflammatory burden, such as HFpEF. Consistent with this concept, subsequent studies have shown that hsCRP levels are not significantly correlated with LVEF, supporting its role as an EF-independent marker of systemic inflammation in HF.^83^

### 2.3 Novel methodologies in biomarker discovery

The discovery of new biomarkers associated with inflammation in HF may be boosted by novel strategic methods. Two emerging approaches are liquid biopsies of extracellular vesicles (EVs) and non-coding RNAs (ncRNAs); interested readers are referred to several comprehensive articles detailing the relevant methodologies and technical considerations.^84–89^

#### 2.3.1 EVs

EVs have recently emerged as both tentative surrogate markers for CV inflammation and paracrine biological shuttle modules with influence in distant target cells.^90,91^ EVs are microvesicles (MVs; small lipid bilayer particles) that bud off from cells following inflammation, cell damage and/or apoptosis. The universal ‛EV’ expression comprises all vesicle subtypes released by activated/damaged cells; they are a highly heterogeneous pool with regard to size, range, origin, content, biochemical and biophysical features, and biological functions. Currently, EVs are classified as apoptotic bodies (ApoBDs), MVs, and exosomes (EXOs). The largest EVs are ApoBDs (∼1 to 5 μm), which are damaged or dead cells in the process of cellular shrinkage and contain intracellular constituents such as DNA or organelles. The smallest EVs are EXOs, which are nanosized cup-shaped vesicles (∼20 to 100 nm) shed by exocytosis of endocytic and intracellular multivesicular bodies upon fusion with plasma membranes. Medium-sized phospholipid vesicles (∼100 nm to 1 μm), which are specifically generated by outward shedding of the plasma membrane through a series of interrelated processes, including enzymatic reactions, cytoskeleton remodelling, and phosphatidylserine exposure, are the most studied in atherosclerotic CVD (ASCVD). These vesicles contain a cargo of membrane skeleton, proteins, lipids, and nucleic acids that are specific to their parental cells.^92^ Given that the molecular signatures of EVs reflect the condition of their parental cells, and that they can be measured using blood samples, EVs are emerging as promising non-invasive biomarker candidates. EVs released by damaged organs to the circulation can facilitate long-distance cell-to-cell communication and mediate organ crosstalk, a phenomenon that has been demonstrated in diverse pathological conditions,^93^ including cancer,^94^ CVD,^95^ and neurodegeneration.^96^ Recently, it has been reported that EVs can facilitate pathological kidney–heart communication and contribute to HF progression through their cargo.^97^ Furthermore, CKD-derived EVs have been shown to induce vascular smooth muscle cell calcification.^98^

#### 2.3.2 ncRNAs

ncRNAs are post-transcriptional regulators of gene expression that do not encode proteins and play critical roles in both cellular homeostasis and disease states. By integrating diverse stress signals, ncRNAs modulate cellular functions and are, therefore, implicated in the regulation of chronic inflammation and the development of HF. Interest in circulating ncRNAs has increased substantially as the search for robust non-invasive biomarkers in HF has intensified. To date, the most commonly investigated types of ncRNAs are the microRNAs (miRNAs) and the long ncRNAs (lncRNAs), which can be found in isolated EVs and in plasma samples.

miRNAs and lncRNAs are secreted by endothelial cells, cardiomyocytes, and fibroblasts, within EXOs or MVs, or bound to lipoprotein complexes, enabling their stable detection in plasma, serum, and urine. Although lncRNAs have been widely investigated in relation to cardiac fibrosis and hypertrophy, the involvement of lncRNAs in inflammatory pathways remains relatively understudied. Notably, ncRNAs exert context-dependent, dual roles in HF pathophysiology, contributing to both tissue injury and repair. Beyond their potential utility as mechanistic biomarkers of organ damage, ncRNAs also represent promising therapeutic targets for modulating the vascular–cardiac axis underlying HF progression.

miRNAs are short RNA sequences (18–22 nucleotides long) that post-transcriptionally regulate and control RNA expression. miRNAs have been shown to modulate gene expression in endothelial function, inflammation, and myocardial hypertrophy.^99^ For example, the miR-223–3p, which was found in EVs in the MERLIN-TIMI 36 trial,^100^ appears to promote pyroptosis, a pro-inflammatory cell-death programme in cardiomyocytes, and to stimulate the release of inflammasome factors.^101^ A regulatory role for miR-223 has been described in the differentiation of granulocytes, dendritic cells and lymphocytes, macrophage polarization, and endothelial and epithelial inflammation.^102^ miR-1 suppresses Krüppel-like factor 4 (KLF4) and activates NF-κB, inducing vascular inflammation and atherosclerosis.^103^ miR-216a promotes inflammation by targeting Smad3 and destabilizing IκBα.^104^ miR-34a-5p activates p53 and p16 pathways to drive endothelial inflammation and senescence.^105^ Low shear stress downregulates miR-181b-5p, thereby relieving STAT3 suppression and activating the NLRP3 inflammasome.^106^ miR-15b-5p metastasis-associated lung adenocarcinoma transcript 1 (MALAT1) sponges miR-15b-5p to upregulate mitogen-activated protein kinase 1 and activate mechanistic target of rapamycin (mTOR), increasing inflammation.^107^ In plasma samples from patients with HF receiving guideline-directed medical therapy, seven miRNAs (let-7a-5p, miR-107, miR-125a-5p, miR-139-5p, miR-150-5p, miR-30b-5p, and miR-342-3p) allowed for differentiation between HFpEF and HFrEF, and between ischaemic and non-ischaemic aetiology, and some clusters showed pathway enrichment analysis on regulation of inflammatory cell transendothelial migration, and inflammatory mediator molecules.^108^ Future studies are needed to investigate whether differential signatures of down-regulated miRNAs in the plasma of patients with HF may help to identify novel cellular mechanisms involved in HF pathogenesis and inflammation.

lncRNAs are non-coding RNA transcripts longer than 200 nucleotides with complex functions, capable of inducing chromatin modifications, altering protein functions, and regulating mRNA stability or processing. lncRNAs typically affect gene expression, alternative splicing, or signalling pathways, and exert their effects via miRNA sponging, epigenetic remodelling, and direct RNA–protein interactions. MIR181A1HG and LINC01235 aggravate inflammasome activation and senescence via NLRP3 and miR-224-3p sponging, while protective lncRNAs, such as MALAT1 and PRKAG2-AS1, mitigate monocyte adhesion and cytokine release through ATG5-dependent autophagy and PRKAG2 stabilization.^109–112^ The endothelial-derived, secreted lncRNAs *Gadlor1* and *Gadlor2* have been found to aggravate cardiac remodelling.^113^

The role of ncRNAs in regulating inflammation in vascular and heart cell function may provide molecular bridges connecting vascular dysfunction to HF and uncover novel diagnostic biomarkers in HF. Furthermore, as our comprehension of ncRNA biology increases, circulating ncRNAs will be instrumental in the development of precision medicine in cardiology, allowing for the earlier detection of HF and optimal stratification of patient HF sub-phenotype.

## 3. hsCRP in CVD and HF

### 3.1 Measuring hsCRP and defining systemic inflammation in CVD

As described, tissue-level mediators including TNF, IL-1β, IL-18, CCL2 (MCP-1), and CXCL8 (IL-8) contribute directly to myocardial inflammation, immune cell recruitment, fibrosis, and remodelling.^114,115^ However, in this review, we emphasize CRP as a well-validated systemic biomarker.

hsCRP is a well-established biomarker of systemic inflammation.^66,116^ Although widely used in clinical practice in the context of acute disease, infections, or sepsis, and therefore not a biomarker specific to CVD, it has been proposed more recently as a risk marker for CV risk assessment. The ACC and the AHA guidelines on primary prevention of CVD and management of blood cholesterol recognize hsCRP ≥ 2 mg/L as a risk marker for CVD,^117,118^ and the 2021 European Society of Cardiology guidelines on CVD prevention in clinical practice consider hsCRP a potential risk modifier, but with limited additional value for risk prediction.^119^ More recently, the 2025 Focused Update of the 2019 ESC/European Atherosclerosis Society Guidelines for the management of dyslipidaemias have recognized persistently elevated hsCRP levels (> 2 mg/L) as risk modifiers for consideration beyond the risk estimation based on the SCORE2 and SCORE2-OP algorithms.^120^ In the Inflammation and Cardiovascular Disease: 2025 Scientific Statement from the ACC, universal hsCRP testing in both primary and secondary CVD prevention is recommended.^116^ In addition, hsCRP has been shown to have incremental prognostic value, beyond NT-proBNP and high-sensitivity troponin T, for the occurrence of HF in patients with adult congenital heart disease,^121^ and as a prognostic marker for mortality in patients with HF, over and above NT-proBNP alone.^122^ The 2004 Centers for Disease Control and Prevention/AHA guidelines on the clinical use of inflammatory markers in patients with CVD recommend that, in metabolically stable patients, two CRP measurements be taken, ideally at least 2 weeks apart, and the results averaged.^123^ The recommended risk categories are low risk, < 1.0 mg/L; average risk, 1.0–3.0 mg/L; and high risk, > 3.0 mg/L. If CRP is greater than 10 mg/L, the test should be repeated and the patient examined for sources of infection or inflammation.^123^ In the published literature, a hsCRP level of ≥ 2 mg/L is most commonly used to define systemic inflammation in CVD. In both clinical trial and real-world populations with established ASCVD, hsCRP ≥ 2 mg/L has been associated with higher risks of HF, peripheral artery disease, stroke, and major adverse CV events (MACE) than hsCRP below this threshold.^124–126^

### 3.2 Systemic inflammation in HF

A link between hsCRP levels and incident HF or risk of CV outcomes in prevalent HF has been reported across HF subtypes. Elevated hsCRP levels have been detected in patient cohorts with both HFpEF (median: 8.54 mg/L) and HFrEF (median: 6.66 mg/L).^73^ Further, in an analysis of patients with established ASCVD from the UCC-SMART cohort in the Netherlands, higher hsCRP was a predictor of incident HFpEF, HFrEF, and HF of unclassified subtype, independent of classical risk factors, including previous myocardial infarction (MI).^127^ A meta-analysis has also indicated that hsCRP is predictive of CV mortality in patients with HFpEF.^128^

In longitudinal studies, elevated hsCRP levels over time have been linked to adverse outcomes. In two analyses from the China PEACE 5p-HF study, patients discharged following HHF with persistently high hsCRP levels had a lower survival rate over the following 12 months than those without.^129,130^ Increasing hsCRP levels over repeated measurements was also associated with an increased risk of incident HF, specifically HFpEF, over a median follow-up of 12 years in an analysis including Black US adults from the Jackson Heart Study.^131^

Several studies have characterized populations with HF based on hsCRP ≥ 2 mg/L. In patients with HFrEF in the DAPA-HF trial, 51% had hsCRP ≥ 2 mg/L, which was associated with higher risks of a composite outcome comprising worsening HF event or CV death, as well as the separate components of this outcome, and with all-cause mortality, compared with hsCRP < 2 mg/L.^49^ Similarly, 53% of patients in a pooled population with HFpEF or HFmrEF from the SELECT, SOUL, and FLOW trials had hsCRP ≥ 2 mg/L,^132^ and in the RELAX trial, 57% of patients with HFpEF had hsCRP ≥ 3 mg/L.^133^ A larger proportion of patients with HFpEF had hsCRP ≥ 2 mg/L in the TOPCAT-Americas sub-study (62%), which was associated with a higher risk of CV death and HHF, compared with hsCRP < 2 mg/L.^134^ The prevalence of hsCRP ≥ 2 mg/L in HF is similar to that observed in populations with ASCVD, estimated to be 35–60%.^125,126,135–137^

## 4. Relationship between comorbidities, other risk factors, and inflammatory pathways in HF

Systemic inflammation is associated with and thought to be driven by various cardiometabolic conditions, such as obesity, type 2 diabetes (T2D), and CKD, which can precede HF or develop in parallel, and contribute to the occurrence of CV events. Chronic inflammatory conditions such as inflammatory bowel disease and RA are also risk factors for incident CVD and HF.^22^ An analysis from the ARIC study supports the mediating role of comorbidities in the relationship between systemic inflammation and HF; high accumulated hsCRP was associated with both HFpEF and HFrEF, but the association was not significant after adjustment for comorbidities,^138^ suggesting that the risk associated with comorbidities is mediated by inflammation.^77,139,140^ To understand the heterogeneity in the inflammatory pathways implicated in HF, it is crucial to consider the relationships between key comorbidities and the different HF subtypes. *Figure 3* summarizes the relationships between comorbidities, inflammation, and HF, in addition to other classic and non-classic risk factors.

**Figure 3:**
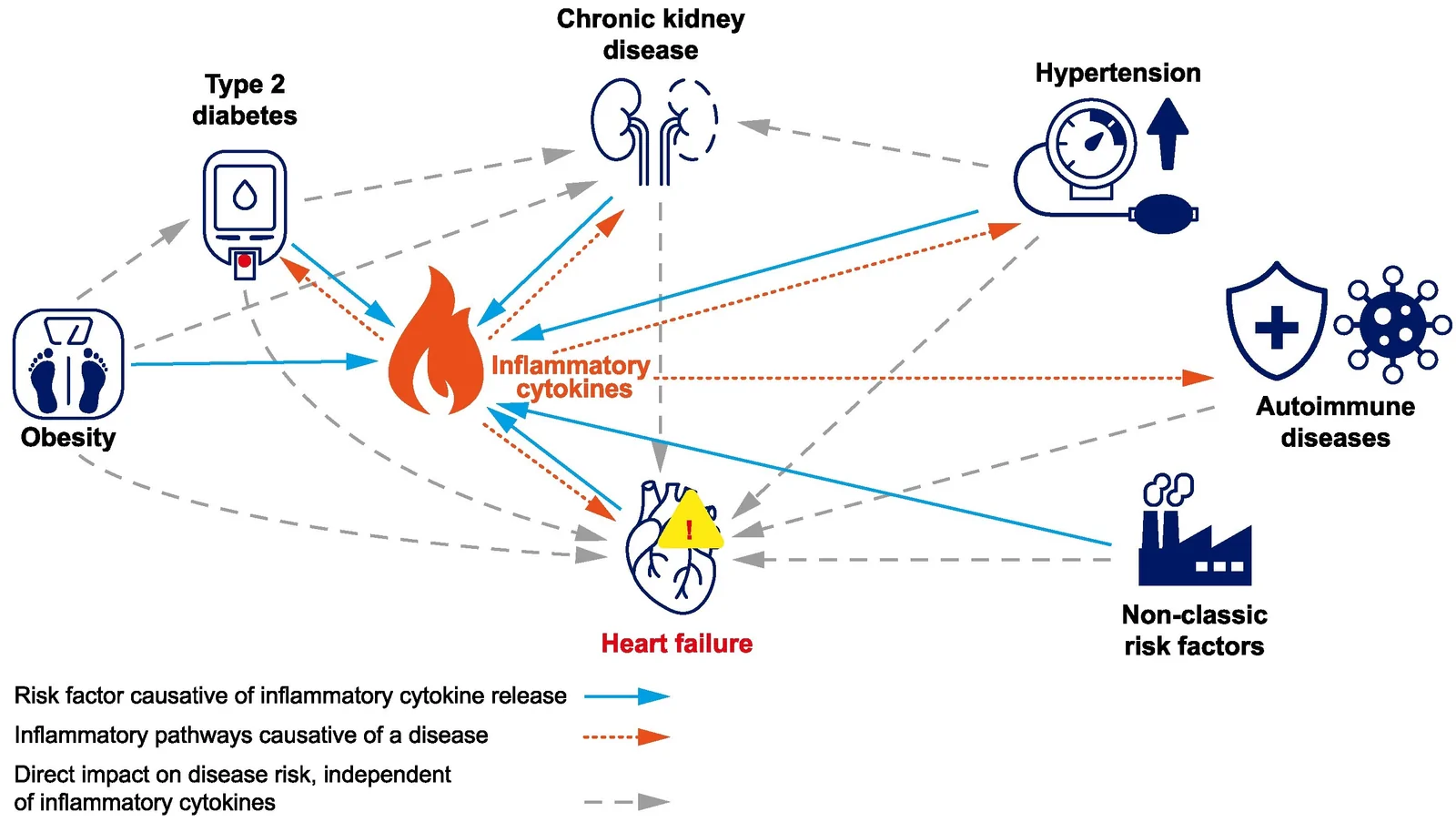
Simplified relationships between key cardiometabolic comorbidities, other risk factors, inflammatory cytokines/pathways, and HF. HF, heart failure.

### 4.1 Obesity

Obesity is common in HF, with a prevalence of up to 84% in HFpEF,^141^ and is a significant risk factor for HFpEF development. Obesity and visceral fat accumulation, as well as epicardial adipose tissue,^78,142^ have multiple pathophysiological effects that contribute to the occurrence of HF.^28^ Among these, visceral adipose tissue can act as a reservoir for adipocytokine production,^143,144^ inducing an inflammatory state that promotes HFpEF via mechanisms including microvascular dysfunction and reduced mitochondrial content and function, which limit oxygen delivery to tissues and aerobic metabolism.^145^ In both HFpEF and HFrEF, secretion of aldosterone and sodium retention combine with increased leptin signalling resulting from obesity, leading to overactivity of neprilysin. This interplay between HF and obesity mechanisms results in cardiac remodelling and fibrosis, as well as adipogenesis and adipocyte dysfunction.^146^ Different metrics used to indicate excess body fat, namely body mass index, waist circumference, and waist-to-hip ratio, have been identified as strong predictors of HFpEF, but not HFrEF.^147,148^ However, obesity may still contribute to HFrEF disease pathology via various mechanisms.^149^ In both the STEP HFpEF (semaglutide vs. placebo) and SUMMIT (tirzepatide vs. placebo) randomized controlled trials, weight loss medications were associated with benefits in terms of reduced HF symptoms and events, and CRP levels in patients with obesity-related HFpEF.^150,151^

### 4.2 Type 2 diabetes

T2D is strongly associated with obesity and is also common in HFpEF, with a reported prevalence of 24–50% in this population.^152–155^ Hyperinsulinaemia and insulin resistance contribute to the occurrence of HF,^156,157^ and T2D is associated with an increased risk of both HFpEF and HFrEF.^158–161^ T2D has distinct roles in promoting the different HF subtypes. T2D is a risk factor for MI, which in turn is a common cause of HFrEF. T2D also has several downstream effects that induce cardiomyocyte death and dysfunction, as described in detail elsewhere.^162^ The state of nutrient excess in T2D leads to downregulation of pathways that promote cellular survival, suppressing autophagy and causing cardiomyocyte stress. In addition, activation of the sodium–hydrogen exchanger leads to deleterious intracellular sodium accumulation, which also results in cardiomyopathy.^157^ The matrix glycoprotein Tenascin-C is upregulated in T2D and drives myocardial dysfunction.^163^ In HFpEF, risks arising from T2D occur in part as a result of visceral and epicardial adipose tissue accumulation, which promotes inflammation and fibrosis.^157^ Comparisons of left ventricular myocardial tissue between patients with HFpEF with T2D and those without T2D have shown relatively higher levels of inflammatory markers and oxidative stress in those with T2D.^164^ A difference in clinical outcomes is also apparent; patients with HFpEF and T2D have a higher disease burden and greater all-cause mortality than those with HFpEF but without T2D.^165^ Diabetes has also been linked to worse outcomes in patients hospitalized for HFrEF.^166^ In a pooled population with HFpEF or HFmrEF from the SELECT, SOUL, and FLOW trials, and in separate HFpEF and HFmrEF subgroups, hsCRP ≥ 2 mg/L was associated with higher HbA1c compared with hsCRP < 2 mg/L.^132^

### 4.3 Chronic kidney disease

Approximately 50% of individuals with HFpEF or HFrEF also have CKD,^167^ and the conditions share risk factors such as T2D, obesity, and hypertension, in the complex continuum of CV-kidney-metabolic (CKM) disease.^168^ The two diseases share haemodynamic, inflammatory, and metabolic pathophysiological drivers, and, notably, reduced glomerular filtration rate and albuminuria, hallmarks of CKD, are also associated with mortality and adverse outcomes in HF.^169^ Individuals with CKD have an approximately threefold higher risk of developing HF than those without CKD,^170^ with slightly higher hospitalization rates for HFpEF than for HFrEF.^171,172^ CKD is also linked to increased risks of mortality and adverse disease outcomes in patients with HF; in fact, in CKD, renal events are much less common than CV and HF events.^173,174^ In a recent characterization of patients with CKD and different HF subtypes, CKD was associated with the highest mortality risk in HFrEF, followed by HFmrEF, then HFpEF, relative to normal kidney function, but conferred similar hospitalization risks.^175^ CKD and HFpEF are linked in a bidirectional manner through multiple pathophysiological processes, including systemic inflammation.^168,169^ CKD is associated with a persistent low-grade inflammatory state,^176^ which can arise via different processes, including overactivation of the mineralocorticoid receptor.^177^ Thus, CKD can result from systemic inflammation and can also trigger inflammatory pathways that contribute to HF pathology.

### 4.4 Hypertension

Hypertension is a key risk factor for HF;^3,178^ more than 50% and up to nearly 90% of patients with HFpEF have been estimated to have hypertension across different studies.^179^ In addition to the left ventricular hypertrophy and diastolic dysfunction induced by hypertension, systemic microvascular endothelial inflammation, fibrosis, and mitochondrial dysfunction are all implicated in the process by which hypertension results in HF.^180^

### 4.5 Autoimmune disorders

Various autoimmune disorders,^181^ including RA,^36,182,183^ psoriasis^184,185^ and systemic lupus erythematosus (SLE)^186^ are associated with a risk of HF as well as death and HHF among patients with HFpEF.^187^ Autoimmune disorders share common, complex immune disturbances, but also have distinct underlying inflammatory axes. The IL-6/JAK-STAT axis is central to RA,^188^ whereas psoriasis is driven by the IL-23/T helper 17 (Th17)/IL-17 axis^189^ and the type 1 interferon (IFN-I) axis is a hallmark of SLE.^190^

RA has been linked to an increased risk of HF, independently of traditional risk factors and intercurrent IHD.^36,182,183^ RA appears to be more strongly linked to HFpEF/HFmrEF than to HFrEF^37,191^ but is associated with an increased risk of death when present in HFrEF.^37^ There is a clear role for inflammation in the relationship between RA and HF: RA patients with higher levels of hsCRP have a greater risk of HF, with a relatively more substantial contribution of hsCRP levels to HFpEF than to HFrEF.^192^ These patients also benefit from anti-inflammatory methotrexate therapy.^192^

Among patients with erythrodermic psoriasis, those with HF have significantly higher hsCRP levels than patients without HF;^193^ other biomarkers of systemic inflammation in psoriasis include components of the NLRP3 inflammasome pathway, such as IL-6 and TNF-α.^194^ Clinical evidence suggests that blocking the inflammatory axis in psoriasis is associated with reduction in the risk of CVD, including HF: use of biologic therapies targeting TNF-α, IL-17, or IL-23 reduced the risk of HF compared with oral anti-psoriatic drugs.^195^ Subgroup analysis from the same study showed that only anti-IL-17 therapy was associated with a significant reduction in CV risk, whereas anti-TNF-α, anti-IL-12/23 or anti-IL-23 agents were not associated with a reduction in risk.^195^ In another study, the benefit in terms of HF risk reduction was greater with inhibition of IL-12/IL-23 with ustekinumab than with anti-TNF-α inhibitors (adalimumab, etanercept, and infliximab).^196^

In SLE, evidence related to the impact of biologics on HF outcomes is limited^197^ and the specific mechanisms underlying the markedly higher prevalence of HF in this population remain to be fully understood.^198^ Traditional CV risk factors cannot explain the high burden of HF in SLE, suggesting a contributing role of inflammatory processes, particularly those mediated by IFN-I, which has both direct and indirect effects on endothelial dysfunction and vascular damage.^198,199^

### 4.6 Non-classic risk factors for HF

There is increasing evidence that non-classic risk factors for HF, such as environmental factors, sleep disturbances, and stress, are mediated through inflammation. Sex-specific risk factors such as polycystic ovary syndrome,^200^ menopause,^201^ gestational diabetes,^202^ and pre-eclampsia^203^ have also been linked to elevated inflammatory biomarkers.

Inflammatory pathways, including the IL-6 and IL-3 signalling pathways, the advanced glycation end products and the receptor for advanced glycation end products (AGE/RAGE) pathway, and the phosphoinositide 3-kinase-protein kinase B (PI3K-Akt) signalling pathway, have been linked to environmental exposures, including built environment, green space, air pollution, temperature, and social vulnerability indicators.^204^ Fine particulate matter air pollution is positively correlated with proinflammatory cytokine levels (e.g. IL-6 and IL-1β), immune cell counts, and markers of endothelial dysfunction.^205^ Air pollutants have also been linked to higher left ventricular mass index^206^ and impaired diastolic function.^207^ There is also a link between diet, inflammation, and HF. In a prospective study that followed approximately 18 000 individuals over a median of 11 years, the highest dietary inflammatory score quartile was independently associated with a 44% higher risk of incident HFrEF, but not HFpEF.^208^ Among patients with established HF, increased intake of trans fatty acids has been positively associated with IL-1β, IL-6, and TNF-α levels after multivariable adjustment including for EF.^209^

Conditions that disturb sleep, including obstructive sleep apnoea and insomnia, have been linked to HF incidence in men independently of established risk factors such as hypertension, diabetes, smoking status, and body mass index.^210,211^ Obstructive sleep apnoea increases the risk of both HFpEF and HFrEF.^212^ Conditions that disturb sleep are also linked to inflammation: sleep deprivation is associated with an increase in inflammatory biomarkers,^213^ and evidence suggests that inflammation mediates the diastolic dysfunction often exhibited by patients with sleep-disordered breathing.^214^ Furthermore, chronic circadian misalignment has been linked to increased TNF-α and CRP levels.^215^

Higher perceived stress is associated with a higher risk of incident HFpEF, but not HFrEF, independent of comorbid conditions.^216^ Acute stress is significantly correlated with increases in circulating IL-6.^217^ In addition, statins, which have anti-inflammatory properties, have been found to attenuate the association between perceived stress and HFpEF.^216^ Symptoms of depression are also associated with an increased likelihood of hospitalization for HFpEF, but not for HFrEF.^218,219^ Among patients with HF, symptoms of depression are associated with higher serum IL-6 and CRP even after adjusting for LVEF, suggesting that the association between depression and systemic inflammation exists across HF subtypes.^220^ In addition, immune dysregulation is linked to higher depressive scores and worse outcomes in HF.^221^ Antidepressant medication has been shown to lower inflammatory biomarkers in patients with HF and depression.^222^

In addition to the underlying comorbidities and risk factors that drive HF, the activation of key neurohormonal systems in established HF, such as the renin–angiotensin–aldosterone system and the sympathetic nervous system, can directly promote cardiac inflammation, contributing to the development of chronic low-grade inflammatory states,^223,224^ referred to as para-inflammation.^115^ Given that para-inflammatory responses exist along a spectrum from subtle, physiological inflammation to full-scale immune activation, gaining a deeper understanding of when and how to intervene is essential. As inflammation plays a central role in the pathophysiology of HF, identifying the tipping point at which it shifts from adaptive to maladaptive is key to developing effective, targeted therapies.^115^

## 5. Inflammatory cytokines in animal models of HF

Experimental studies in animals have demonstrated that activation of proinflammatory cytokine and immune signalling pathways is associated with cardiac remodelling and dysfunction, and that modulation of these pathways can attenuate disease phenotypes in selected models (*Figure 4*).^225–227^ However, despite a strong preclinical rationale, phase 3 clinical trials of anti-inflammatory strategies have largely failed to meet their primary endpoints and, in some cases, have resulted in worsened HF outcomes or increased mortality.^115^ This discrepancy underscores the need to evaluate the strengths and limitations of animal models carefully, particularly given that proinflammatory cytokines may act as biomarkers, pathological modulators, or context-dependent mediators of adaptation rather than universal causal drivers of HF progression.

**Figure 4:**
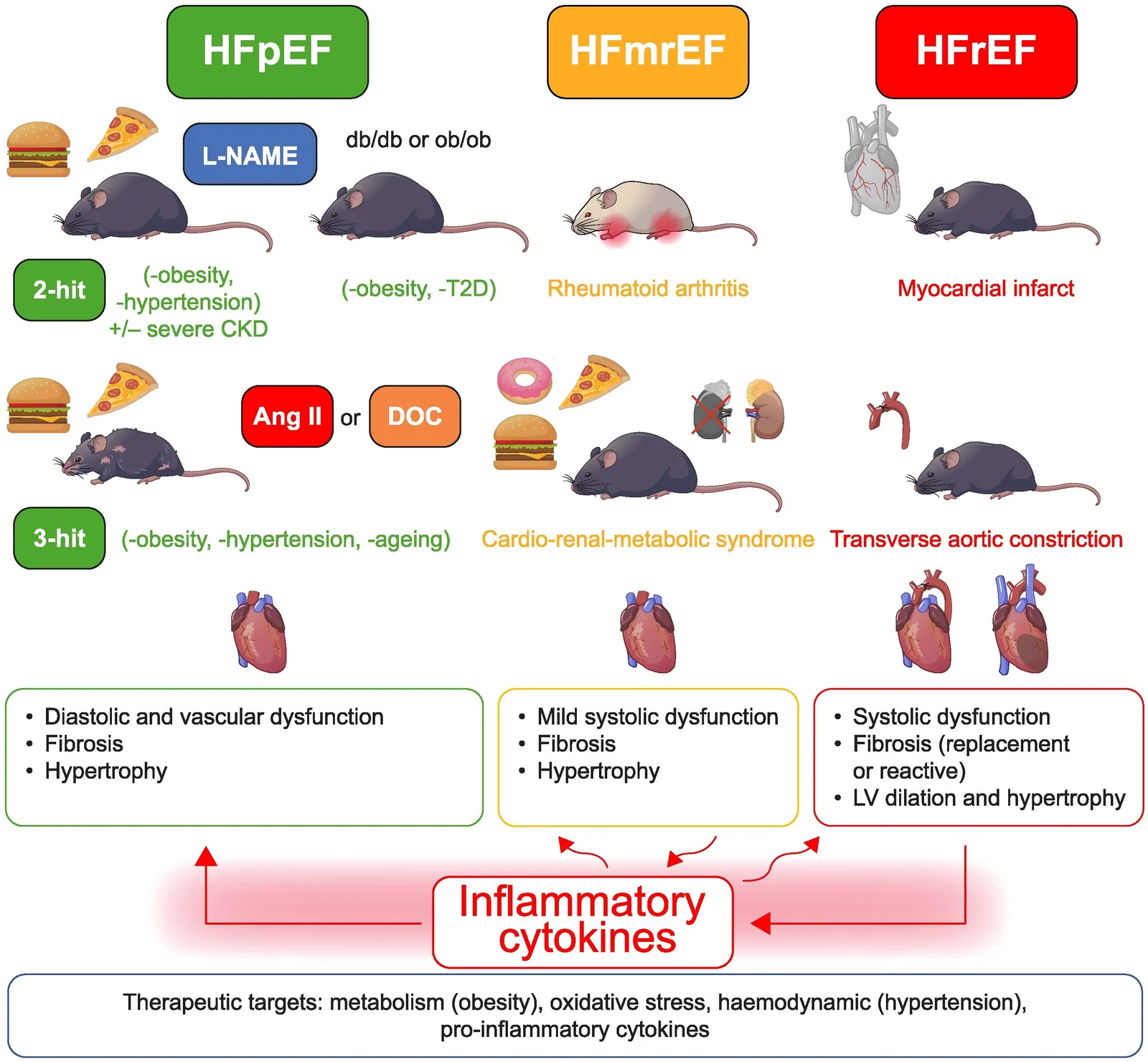
Mouse models of HF subtypes. Figure developed using BioRender. Ang II, angiotensin II; CV, cardiovascular; DOC, deoxycorticosterone; HF, heart failure; HFmrEF, heart failure with mildly reduced ejection fraction; HFpEF, heart failure with preserved ejection fraction; HFrEF, heart failure with reduced ejection fraction; L-NAME, nω-nitro-L-arginine methyl ester; LV, left ventricular; T2D, type 2 diabetes.

Numerous small animal models have been developed to study HF pathophysiology and therapeutic strategies. Most mouse studies assess systolic function using fractional shortening and apply a cut-off of 40% to indicate dysfunction. Ejection fraction can also be calculated from left ventricular dimensions, but normal ejection fraction values in mice are generally higher than in humans (in healthy mice, ejection fraction can range from about 60–90%), which limits the interpretation of the results if raw ejection fraction values are compared between mice and humans.^228^ Importantly, inflammatory states may differentially affect myocardial mechanics, diastolic function, and myocardial energetics before overt reductions in fractional shortening or EF become apparent.^115,229^ Accordingly, additional functional and mechanistic approaches are increasingly employed in animal models of inflammation-associated HF, including myocardial strain analysis (e.g. global longitudinal strain),^230^ Doppler echocardiography to assess diastolic dysfunction,^228,231^ invasive pressure–volume loop analysis for load-independent assessment of systolic and diastolic performance,^228,232^ and cellular and molecular assays that interrogate inflammatory signalling, fibrosis, oxidative stress, and altered calcium handling.^114,233^ Together, these complementary techniques provide a more nuanced assessment of cardiac dysfunction in the setting of systemic inflammation.

These models rely on a range of approaches, including a combination of high-fat diet (HFD) and vascular stressors (e.g. mineralocorticoids, angiotensin II, nitric oxide synthase (NOS) inhibitor nω-nitro-L-arginine methyl ester [L-NAME]), genetic manipulation (ob/ob or db/db mice to mimic T2D) or surgical techniques [e.g. MI or transverse aortic constriction (TAC)]. The ‘multiple-hit’ approach is often employed to model the multifactorial nature of HFpEF, in which comorbidities such as hypertension, obesity, and metabolic syndrome accelerate disease progression.^234,235^

HFpEF models aim to capture diastolic dysfunction, remodelling, and immune and cytokine-associated interstitial fibrosis, which increase myocardial stiffness and contribute to disease pathogenesis.^236^ A HFpEF model induced by a combination of metabolic and hypertensive stressors (HFD plus L-NAME) replicates many systemic and cardiac features characteristic of human HFpEF,^234,235^ with a novel inflammatory-nitrosative mechanism, in which iNOS-induced nitrosative stress disrupts endoplasmic reticulum stress signalling via IRE1α–XBP1. Thus, targeting this axis may offer therapeutic potential.^234,235^ The 2-hit model (HFD plus L-NAME) has been further intensified by subtotal nephrectomy (severe surgical reduction of renal mass) and retinol treatment, resulting in persistent renal dysfunction, sustained uraemic and inflammatory stress, and strong activation of profibrotic pathways, including JAK2-STAT5 signalling.^237^ This combined metabolic, vascular, and renal injury drove concentric hypertrophy, interstitial fibrosis, and diastolic dysfunction with preserved EF.^237^

Interestingly, in other preclinical models of HFpEF, genetic disruption of IL-1β protected HFpEF mice from pathogenesis by modulating inflammation and endoplasmic reticulum stress, likely through regulation of these pathways.^238^

Ageing is a critical driver of HFpEF, especially when combined with hypertension, obesity, and vascular disease.^239^ In a preclinical ‘3-hit’ HFpEF model, adult mice of both sexes were fed a HFD for 13 months, with deoxycorticosterone added in the final month. This combination induced pathological cardiac hypertrophy, hypertension, vascular dysfunction, diastolic impairment, oxidative stress, NLRP3 activation, and reduced exercise capacity.^240^ Treatment with β-hydroxybutyrate mitigated HFpEF features by disrupting the cycle between mitochondrial dysfunction and inflammation, via anti-inflammatory and mitochondrial-stabilizing effects rather than acting as an alternative energy source.

In another HFpEF model using aged female mice fed a HFD and treated with angiotensin II, the therapeutic effect of semaglutide was evaluated.^241^ The results showed that weight loss alone was insufficient for cardioprotection, highlighting the need for therapies that target metabolic, inflammatory, and haemodynamic pathways beyond body mass reduction.

Transgenic mice with defective leptin (ob/ob) or leptin receptor (db/db) are widely used in preclinical studies to mimic obesity and T2D.^242^ These mice have features resembling HFpEF.^243–246^ Notably, db/db mice show a more severe phenotype than ob/ob mice, likely due to higher levels of pro-inflammatory cytokines (e.g. IL-6) in adipose tissue and greater macrophage infiltration^242^

In a late-stage RA model, increased cardiac mRNA levels of pro-inflammatory cytokines were associated with mildly reduced systolic function, impaired excitation-contraction coupling in cardiomyocytes, and cardiac remodelling (hypertrophy and fibrosis).^247^ During acute RA, isolated cardiomyocytes exhibited decreased Ca^2+^ sensitivity but enhanced Ca^2+^ transients, preserving contractility.^248^ Thus, the impact of inflammation on CV function might depend on the duration of cytokine exposure and lead to molecular adaptations, such as post-translational modifications of Ca^2+^ handling proteins.^247,248^

Models of CKM syndrome combining surgical and metabolic stressors with unilateral nephrectomy and a high-salt-sugar-fat diet in mice have also been reported. These mice present mildly reduced systolic function and a significant increase in circulating IL-6 levels.^247,249^ However, HFmrEF remains poorly defined in preclinical literature, representing a significant gap that limits mechanistic insights and the development of targeted therapies.

Preclinical models of HFrEF induced by surgical interventions reproduce hallmark features of HFrEF, including systolic dysfunction and remodelling.^236,250,251^ These include cardiac pressure overload via TAC or MI induced by either permanent coronary artery (CA) ligation or transient ischaemia–reperfusion (I/R) injury, for a duration of 4–6 weeks. MI induced by permanent CA ligation produces a more severe phenotype than TAC, with greater replacement fibrosis rather than reactive fibrosis.^252^ In contrast to acute inflammatory responses typically studied at ≤24 h after I/R, several murine studies have demonstrated that I/R injury can lead to sustained structural remodelling and persistent immune cell infiltration over weeks.^253^ Thus, I/R injury is associated with a distinct inflammatory profile marked by an abrupt and intense innate immune response driven by reperfusion-associated oxidative stress, neutrophil infiltration and rapid inflammasome activation, followed by a more pronounced resolution and reparative phase compared with permanent ischaemia.^114,253^ In both MI and TAC models, inflammation is initiated locally within the myocardium in response to tissue injury and mechanical stress, leading to activation of innate immune pathways, including NLRP3 inflammasome assembly and downstream cytokine production. However, the timing, magnitude, and cellular composition of the inflammatory response differ between permanent ligation and I/R models, with important implications for post-injury remodelling and therapeutic targeting.^254^ Targeting the inflammasome, particularly at the right post-injury time window, may reduce inflammation-driven remodelling and prevent HF progression. Among these mediators, IL-1β has emerged as a key effector cytokine linking early sterile inflammation to adverse remodelling and contractile dysfunction. Experimental studies in murine MI models demonstrate that IL-1β signalling directly impairs systolic function, promotes cardiomyocyte apoptosis and exacerbates ventricular dilation, whereas pharmacological or genetic inhibition of IL-1β or its receptor attenuates inflammation-driven remodelling and improves cardiac function.^255–258^ Recent findings showed that dapagliflozin treatment ameliorated the CV phenotype in both MI and TAC mice by inhibiting macrophage-mediated inflammation and myofibroblast activation.^259^ Likewise, a recent study demonstrated that dietary nitrate supplementation in early-stage HFrEF, induced by permanent CA ligation MI or TAC, reduced inflammation by promoting macrophage polarization from the pro-inflammatory M1 to the anti-inflammatory M2 phenotype, thereby improving haemodynamic function and attenuating adverse cardiac remodelling.^260^

Studies in both canine and rodent models showed that circulating TNF exerts direct adverse inotropic effects, both *in vivo* and in isolated cardiac tissue.^261^ Cardiac TNF overexpression resulted in depressed systolic function in a gene dose-dependent manner.^262^ However, TNF also has a cytoprotective role. Global deletion of the TNF receptor resulted in increased infarct size after MI,^263^ whereas TNF overexpression prevented functional deterioration in dilated cardiomyopathy models.^264^ These findings suggest a dual role for TNF: detrimental in chronic remodelling while potentially supporting early healing after myocardial injury. Thus, inflammatory cytokines can exert both maladaptive and compensatory effects depending on timing, magnitude, cellular context, and disease stage. Although mouse models have provided important mechanistic insights into inflammation-driven HF, major limitations constrain their translational value. Many models rely on young, genetically homogeneous animals and short-term injury paradigms that do not fully reflect the chronic, comorbidity-driven and age-dependent nature of human HF, although important exceptions include emerging aged and multi-hit HFpEF models. Moreover, inflammatory responses in mice are often exaggerated, temporally compressed and differ in immune cell composition and cytokine signalling from those in humans, which can lead to overestimation of therapeutic efficacy. In addition, surgical models such as MI or TAC primarily reflect localized myocardial injury rather than the systemic inflammatory burden typical of clinical HF. Nevertheless, animal models remain indispensable for defining causal immune mechanisms, dissecting temporal and cell-specific inflammatory pathways, and testing targeted interventions under controlled conditions that are not feasible in humans, thereby providing a critical foundation for rational translational trial design.^114,115^ Looking ahead, integrating detailed functional and physiological phenotyping with molecular and cellular analyses will be critical to more precisely link inflammatory effectors to HF progression and therapeutic response.

## 6. Inflammatory pathways—treatment implications

### 6.1 Anti-inflammatory effects of approved therapies

Heterogeneous disease aetiologies, comorbidities, and inflammatory pathways are implicated in HF, highlighting the multiplicity and complexity of potential therapeutic targets. Many current approved treatment options for HF have pleiotropic effects, including anti-inflammatory effects. In a systematic review and meta-analysis of randomized controlled trials, ACEIs reduced levels of CRP, IL-6, and TNF-α; angiotensin receptor blockers reduced only IL-6.^265^ The beta-blockers bisoprolol and carvedilol have also been associated with significant reductions in hsCRP in patients with HF.^266^ Preclinical studies in rodents have suggested that SGLT2 inhibitors may exert anti-inflammatory effects;^267^ however, clinical data from the EMPIRE-HF^268^ and DAPA-HF^49^ randomized controlled trials have not conclusively confirmed this. As previously highlighted, glucagon-like peptide-1 receptor agonists (GLP-1RAs) reduce CRP levels.^269^ There is also some evidence that lifestyle changes as part of HF treatment may reduce inflammation. In several studies identified in a systematic review, physical training was associated with reduced TNF-α levels in patients with HF.^270^ The COVID-19 pandemic underscored the substantial CV risks associated with viral respiratory infections, including increased rates of HF and MACE, particularly amongst populations at high risk. Respiratory infections such as influenza activate pro-inflammatory and prothrombotic pathways that destabilize atherosclerotic plaques and impair cardiac function, leading to increased myocardial stress and oxygen demand, and potential ischaemia, arrhythmias, HF decompensation, and/or inflammatory myocardial injury. Emerging evidence highlights vaccination as an effective preventive strategy to mitigate these risks, supporting its integration as a core component of CVD prevention.^271^

Some treatments are known to have differential effects in HFpEF and HFrEF, and treatment decisions and future trial designs must be considered carefully. Statins are effective in the prevention of incident HF,^272^ but in established HF their effects differ between HFpEF, in which statin therapy is associated with reduced risks of mortality and CV outcomes,^273^ and HFrEF, in which statins appear to improve these outcomes only in patients with IHD.^274^ The efficacy of statins is linked to their known pleiotropic, anti-inflammatory effects.^275^ In the CORONA trial of rosuvastatin, improved outcomes were observed in patients with hsCRP ≥ 2 mg/L but not those with hsCRP < 2 mg/L.^276^ This contrasts with the JUPITER randomized placebo-controlled trial, in which all included patients had hsCRP ≥ 2 mg/L, and a significant reduction in MACE was observed with rosuvastatin.^277^ GLP-1RAs can reduce the occurrence of atherosclerotic events in HFpEF, but in HFrEF their use should be considered with caution owing to a potential link with worsening CV events and arrhythmias.^278^

### 6.2 Development of agents targeting inflammatory pathways

Despite guideline-directed medical therapy, systemic inflammation often persists in patients with established ASCVD and is associated with a sustained risk of recurrent CV events in this population.^279^ Such individuals may benefit from existing or future therapies that target systemic inflammation directly (*Figure 5*). Many studies have investigated therapies targeting different inflammatory cytokines across the range of LVEF subpopulations and varying clinical presentations. However, phase 3 trials that translate these findings into the clinical arena have been of limited value.^280^ Although anti-TNF-α agents (infliximab and etanercept), first investigated in the 1990s, showed neutral findings and even signals for harm in HF,^281,282^ the observed improvements in vascular and LV function using anakinra in patients with RA sparked interest in this recombinant human IL-1 receptor antagonist for the treatment of HF.^283^ Small pilot trials involving patients with HFpEF and elevated hsCRP levels yielded conflicting, albeit hypothesis-generating results: while hsCRP and NT-proBNP levels decreased with anakinra, significant improvements in aerobic exercise capacity with anakinra compared with placebo were observed in DHART but not in DHART2.^284,285^ The REDHART trial also demonstrated that longer anakinra treatment durations and administration during a more stable phase following an acute decompensated HF (ADHF) event provided greater benefits than a shorter treatment duration with anakinra or placebo.^286^ However, in the phase 2 REDHART2 trial, longer anakinra treatment duration (i.e. 24 weeks) did not significantly affect peak oxygen consumption compared with placebo in patients with HFrEF and ADHF.^287^

**Figure 5:**
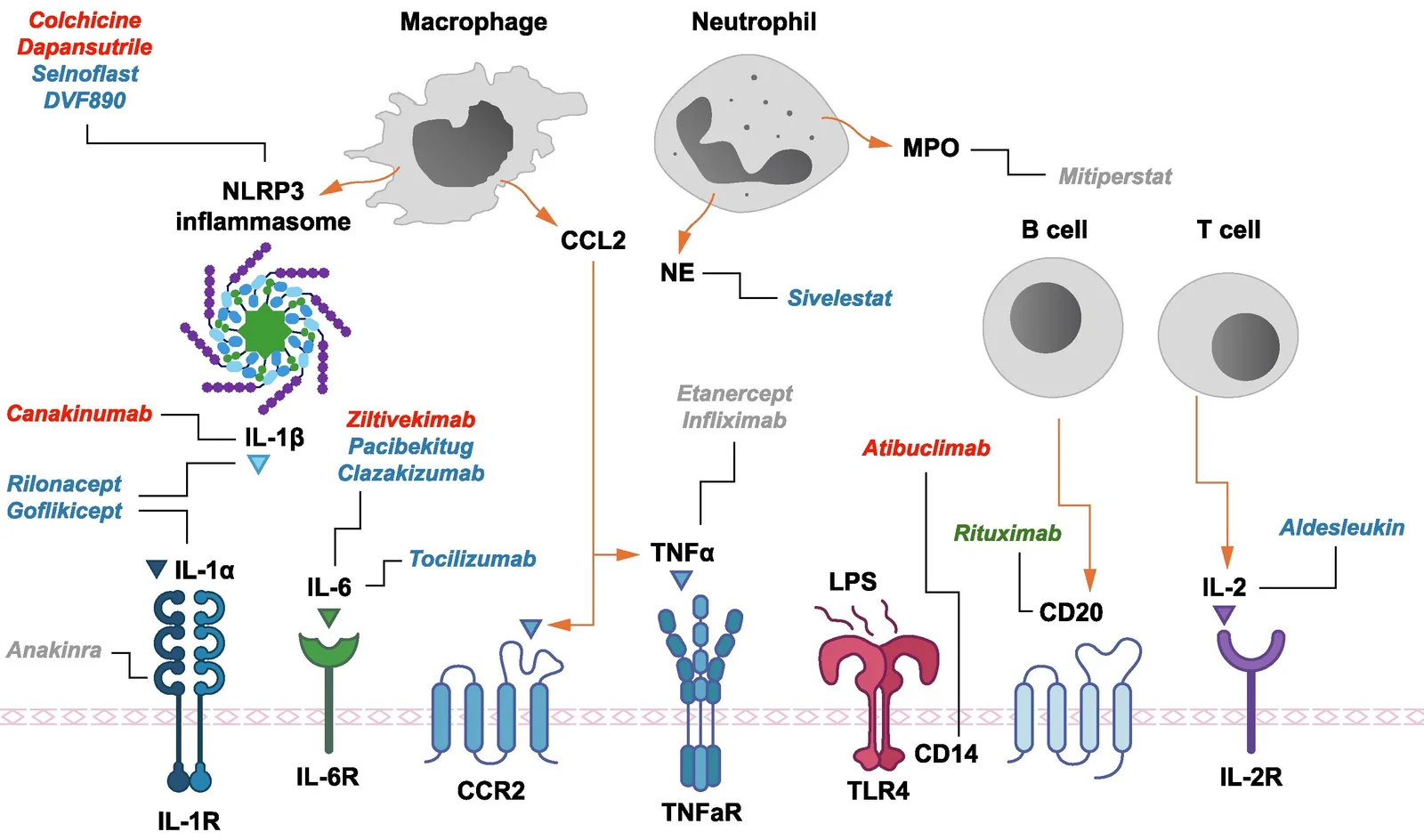
Summary of investigated drugs and those under investigation. Figure developed using BioRender. Grey indicates drugs that have been investigated for HF and had negative results. Red indicates drugs that are under investigation for HF. Blue indicates investigational drugs that have not yet been extensively evaluated in dedicated HF populations. Green indicates rituximab, which has a case series in dilated cardiomyopathy. Other interleukin-targeting therapies that have not yet been extensively evaluated in dedicated HF populations and are not shown in the figure include olokizumab, siltuximab, secukinumab, ixekizumab, brodalumab, ustekinumab, and guselkumab. CCL2, C-C chemokine ligand 2; CCR2, C-C chemokine receptor type 2; HF, heart failure; IL, interleukin; LPS, lipopolysaccharide; MPO, myeloperoxidase; NE, neutrophil elastase; NLRP3, nucleotide-binding oligomerization domain-like receptor family pyrin domain containing 3; R, receptor; TLR, Toll-like receptor; TNF, tumour necrosis factor.

Although the myeloperoxidase inhibitor, mitiperstat, significantly attenuated myeloperoxidase activity in the phase 2a SATELLITE study,^62^ the phase 3 ENDEAVOR trial comparing the effect of mitiperstat vs. placebo in patients with stable, symptomatic HFpEF (albeit without use of enrichment criteria for systemic inflammation) did not find significant differences for its dual primary endpoints of Kansas City Cardiomyopathy Questionnaire (KCCQ) total symptom score or 6-min walk distance.^288^ The phase 2 MYSTERY-HF trial yielded similar results in patients with HFrEF: compared with placebo, mitiperstat did not affect NT-proBNP levels, left ventricular remodelling, KCCQ total symptom score, or 6-min walk distance.^289^

## 7. Outlook

### 7.1 Emerging treatments

Several new molecules that specifically target relevant inflammatory pathways are under development for the treatment of HF. A small phase 1/2 trial (NCT06556810) is investigating atibuclimab (IC14), an anti-CD14 monoclonal antibody, for the treatment of adults hospitalized with ADHF.^290^ Targeting of CD20 has also been reported: in a case series of six patients with CD20+ inflammatory dilated cardiomyopathy and EF of 14–45% at baseline, treatment with rituximab improved EF and New York Heart Association class and reduced NT-proBNP levels.^291^

Exploratory analyses from the CANTOS trial demonstrated that IL-1 blockade with canakinumab was associated with a reduction in HF-related event rates^292^ and improved exercise capacity,^293^ further supporting the potential benefits of anti-cytokine therapy in HF. The first randomized trial evidence demonstrating significant risk reductions in HF event rates (HHF and the composite of HHF or HF-related death) following anti-inflammatory therapy targeting the NLRP3-to-IL-1β-to-IL-6-to-CRP pathway of innate immunity came from an exploratory analysis of the CANTOS trial.^292^ CANTOS tested canakinumab (a monoclonal antibody against IL-1β) vs. placebo amongst approximately 10 000 patients with a previous MI and elevated hsCRP.^292^ In CANTOS, baseline levels of increasing IL-6 and hsCRP independently predicted incident HHF.^292^ Importantly, participants achieving on-treatment hsCRP concentrations < 2 mg/L derived the greatest risk reductions in outcomes.^292^

Despite the fact that high-dose prednisone data from previous studies showed a lack of benefit and important safety concerns,^294^ prednisone therapy was recently re-evaluated in CORTA-HF;^295^ a small, open-label pilot study with mixed results that warrant further evaluations, some of which are ongoing. Colchicine, a well-tolerated and inexpensive drug with a main mechanism of action involving the inhibition of microtubule assembly, has also garnered considerable interest as a potential anti-inflammatory agent in HF. Despite the neutral results published by Deftereos *et al.*^296^ in patients with stable HFrEF, several ongoing trials are testing the potential benefits of colchicine on CV event reduction, microvascular dysfunction, and exercise capacity and quality of life improvement across the whole spectrum of LVEF. The COLICA trial in patients with ADHF showed that 8 weeks of 0.5 mg of colchicine twice-daily vs. placebo was well tolerated and effective in reducing systemic inflammation, but demonstrated no differences in NT-proBNP levels or clinical outcomes/events.^297^ A phase 1b study of dapansutrile (NCT03534297), another NLRP3 inflammasome inhibitor, was completed in 2019 and demonstrated that dapansutrile was well tolerated in patients with HFrEF; however, no further investigations have been launched since.^298^

Ziltivekimab is a narrow-spectrum, human monoclonal antibody directed against the IL-6 ligand. Unlike other clinically available IL-6 signalling inhibitors for rheumatologic and inflammatory conditions, ziltivekimab was specifically developed for atherosclerotic diseases associated with an IL-6-linked pro-inflammatory response, including HF.^299^ Because it is a monoclonal antibody targeting the IL-6 ligand rather than the IL-6 receptor, ziltivekimab, when used at lower doses, may have fewer adverse effects on the lipid profile, haematological parameters, and liver function than other IL-6 agents with regulatory approval, from which it differs mechanistically (e.g. tocilizumab and sarilumab); however, some uncertainties remain. This approach may be less advantageous when the concentration of circulating IL-6 is very high, because ziltivekimab may be unable to fully saturate and neutralize all free IL-6. In addition, the long-term consequences of sustained IL-6 suppression in HF, including potential effects on host defence and tissue repair, remain incompletely understood.

In the phase 2 RESCUE trial,^300^ 24-week treatment with ziltivekimab, administered subcutaneously once-monthly, in patients with stage 3–5 CKD and systemic inflammation (hsCRP ≥ 2 mg/L) showed significant reductions in hsCRP ranging from 77 to 92% across the ascending ziltivekimab dose range (i.e. 7.5, 15, and 30 mg), compared with –4% with placebo; approximately twice the hsCRP inhibition seen with canakinumab in CANTOS. In addition, the 15 and 30 mg doses of ziltivekimab showed minimal between-participant variation in hsCRP reduction, suggesting that targeting the more downstream IL-6 ligand with ziltivekimab (as opposed to the more upstream IL-1β) may reduce the variation in on-treatment hsCRP reductions seen in CANTOS with canakinumab.^299^ The HERMES and ATHENA trials are evaluating ziltivekimab for CV outcomes and functional outcomes, respectively, in patients with HFmrEF or HFpEF and systemic inflammation.^301^

Other interleukin-targeting therapies have not yet been extensively evaluated in dedicated HF populations. These include IL-6 pathway inhibitors (e.g. clazakizumab, pacibekitug, olokizumab, siltuximab), IL-1 inhibitors such as rilonacept and goflikicept, IL-2 inhibitors (e.g. aldesleukin) and agents targeting the IL-17 and IL-12/23 pathways (e.g. secukinumab, ixekizumab, brodalumab, ustekinumab, and guselkumab), which have primarily been studied in rheumatologic and dermatologic conditions. While some IL-6 inhibitors, such as tocilizumab, have been investigated in cardiovascular settings (e.g. acute coronary syndromes), their role in HF remains unclear. Similarly, NLRP3 inflammasome inhibitors (e.g. selnoflast and DFV890) are currently being explored in inflammatory and metabolic diseases, but have not yet been evaluated in HF-specific clinical trials.

## 8. Conclusion

In this review, we have highlighted the heterogeneity of inflammatory pathways in HF and distinguished between the inflammatory profiles of HF subtypes. Inflammation is relevant across all HF subtypes, and systemic inflammation, defined as hsCRP ≥ 2 mg/L, is prevalent and associated with adverse outcomes in both HFpEF and HFrEF; however, differing biomarker profiles across subtypes suggest disparities in underlying processes. HFmrEF is more clinically similar to HFrEF than to HFpEF, but the role of inflammatory cytokines in HFmrEF remains poorly understood, and further research into this subtype is needed. Emerging approaches such as EVs and ncRNAs may facilitate the discovery of novel inflammatory biomarkers specific to HF subtypes. Comorbidities such as obesity, T2D, and CKD can be both causative of and worsened by inflammation, and have complex, bidirectional interrelationships with each HF subtype. Thus, inflammation is often the mediator via which comorbidities drive HF, as demonstrated by multiple animal models of HF subtypes and relevant comorbidities. Although various approved treatments for HF have moderate pleiotropic anti-inflammatory effects, therapies developed to modulate inflammation have largely produced mixed or negative results in clinical trials to date. Ongoing trials are investigating the potential of new treatments targeting inflammatory cytokines to improve outcomes in HF. Crucially, further work is warranted to develop effective, targeted therapies that account for the heterogeneity of inflammation in HF.

## Authors’ contributions

All authors participated in the conceptualization, writing, and critical revision of the text.

## Data Availability

The authors confirm that no novel data are included in this analysis, and all information and data discussed are supported by the references.
